# Does concomitant acromioplasty facilitate arthroscopic repair of full-thickness rotator cuff tears? A meta-analysis with trial sequential analysis of randomized controlled trials

**DOI:** 10.1186/s40064-016-2311-5

**Published:** 2016-05-21

**Authors:** Lei Song, Ling Miao, Peng Zhang, Wen-Liang Wang

**Affiliations:** Postgraduate Training Base, Affiliated Hospital of Logistics University of the Chinese People’s Armed Police Forces, Liaoning Medical University, No. 220, Chenglin Road, Hedong District, Tianjin, 300162 China; Department of Orthopaedic Center, Affiliated Hospital of Logistics University of the Chinese People’s Armed Police Forces, No. 220, Chenglin Road, Hedong District, Tianjin, 300162 China

**Keywords:** Acromioplasty, Subacromial decompression (SAD), Rotator cuff, Repair, Arthroscopic, Meta-analysis, Randomized controlled trials (RCTs)

## Abstract

**Purpose:**

To conduct a meta-analysis with randomized controlled trials (RCTs) published in full text to determine the benefits of concomitant acromioplasty in repairing full-thickness rotator cuff tears.

**Methods:**

Literature search was performed in PubMed, Embase and the Cochrane Library from databases inception through February 2016 to identify RCTs evaluating the efficacy of performing a concomitant acromioplasty. Statistical heterogeneity among studies was quantitatively evaluated by I-squared index (I^2^) and trial sequential analysis (TSA) was applied to control random errors.

**Results:**

Five RCTs totaling 523 patients were included. There was no statistically significant difference in Constant score (WMD = 1.00; 95 % CI −4.40 to 6.41; P = 0.72), University of California-Los Angeles (UCLA) score (WMD = 0.48; 95 % CI −0.79 to 1.76; P = 0.46), visual analog scale (VAS) for pain (WMD = −0.23; 95 % CI −0.58 to 0.11; P = 0.19) and re-tear rate (RR = 0.46; 95 % CI 0.14 to 1.53; P = 0.21) between acromioplasty group and the nonacromioplasty group. However, it was found to be related to a greater increase in American Shoulder and Elbow Surgeons (ASES) score (WMD = 3.02; 95 % CI 0.24 to 5.80; P = 0.03). Unfortunately, this difference was not reinforced by subsequent TSA. In addition, subgroup analysis showed no substantial difference of ASES score in patients with type-1 (WMD = −8.21; 95 % CI −23.55 to 7.14; P = 0.29), type-2 (WMD = 0.97; 95 % CI −5.10 to 7.05; P = 0.75), or type-3 (WMD = 2.32; 95 % CI −9.96 to 14.61; P = 0.71) acromion.

**Conclusions:**

A significant higher ASES score was observed during the comparison despite lacking reinforcement by TSA. No difference was found in Constant score, UCLA score, VAS, re-tear rate and subgroup analysis did not confirm the impact of acromion type on eventual therapeutic outcome. Future studies with large number of participants, long-term follow-ups, data of patient-reported outcomes and stratification for acromion type are of the essence for demonstrating whether functional or structural differences exist in patients undergoing arthroscopic repair of full-thickness rotator cuff tears with or without acromioplasty.

## Background

Since initially presented its successful utilization in treating chronic impingement syndrome by Neer (1972), open anterior acromioplasty had brought numerous preferable clinical outcomes (McShane et al. 1987; Hawkins et al. 1988; Bigliani et al. 1989). Furthermore, Ellman made arthroscopic acromioplasty popular in 1987 (Ellman 1987). Therefore, acromioplasty has gradually become one of the most frequently performed orthopaedic procedures over the last few decades (Vitale et al. 2010; Yu et al. 2010). More recently, Paloneva et al. (2015) stated that acromioplasty was the most common concomitant procedure in Finland, performed in nearly 40 % of rotator cuff repairs in 2011. Technically speaking, Acromioplasty is simple and it is often served as a component part of subacromial decompression (SAD), which also contains release of the coracoacromial ligament (CAL) and subacromial bursectomy as well (Ellman 1987).

Nevertheless, the benefits of acromioplasty in repair of rotator cuff tears remain controversial regarding to its therapeutic value. Several studies presented the association between rotator cuff problem and acromial shape (Bigliani et al. 1991; Balke et al. 2013), and a type-3 hooked acromion was found vulnerable to rotator cuff disease. Additionally, acromial spurs at the anterior and lateral edges are associated with full-thickness rotator cuff tears (Nyffeler et al. 2006; Ames et al. 2012; Fujisawa et al. 2014). Under Neer’s extrinsic theory, acromioplasty was advocated to modify acromial morphology, reducing extrinsic compression on the rotator cuff (Neer 1972), improving arthroscopic visualization during rotator cuff repair (Shi and Edwards 2012; Frank et al. 2014), and inducing a healing response through bleeding bone in the subacromial space (Neer 1972; Randelli et al. 2009; Shi and Edwards 2012). However, proponents of the intrinsic theory argued against acromioplasty for preservation of the CAL and deltoid attachment, the economics of saved operative time and equipment, and the neurobiology of subacromial space (Shi and Edwards 2012).Two studies (Goldberg et al. 2001; Matsen 2009) consecutively substantiated that acromioplasty was not necessary for successful repair of full-thickness rotator cuff tears. Thus, the debate was more urgent to be settled.

To date, several reviews (Nottage 2003; Shi and Edwards 2012; Frank et al. 2014; Chintanpreet Singh and Murrell 2015; Familiari et al. 2015) published to confirm the role of acromioplasty in rotator cuff repairs have suggested that routine use of concomitant acromioplasty is not supported by current evidence. Based on three published RCTs (Gartsman and O’connor 2004; Milano et al. 2007; MacDonald et al. 2011) and one conference paper abstract, a previous level I systematic review and meta-analysis (Chahal et al. 2012) was conducted to assess the role of SAD in patients undergoing arthroscopic repair of full-thickness tears of the rotator cuff, manifesting that there was no statistically significant difference after a quantitative synthesis of available data in 373 patients. But, several limitations could be found in this meta-analysis such as the pooled evidence which was only available for ASES and Constant scores as well as re-tear rate, inconsistencies between characteristics counting and data synthesis. Subsequently, two updated high-quality RCTs (Shin et al. 2012; Abrams et al. 2014) were published in 2012 and 2014, which allowed for more evidence pooling. Recently, another meta-analysis (Meena and Gangary 2015) including five RCTs (Gartsman and O’connor 2004; Milano et al. 2007; MacDonald et al. 2011; Shin et al. 2012; Abrams et al. 2014) was published, but unfortunately, there was some errors in data extraction.

Consequently, it is of necessity to conduct a latest and more persuasive meta-analysis that contains all full-text published RCTs (Gartsman and O’connor 2004; Milano et al. 2007; MacDonald et al. 2011; Shin et al. 2012; Abrams et al. 2014) to evaluate the efficiency of applying acromioplasty in conjunction with arthroscopic rotator cuff repair. We made the present meta-analysis to complete the evaluation mentioned above. Predefined primary outcome was the available functional scores. Secondary outcome was the incidence of postoperative re-tear. Tertiary outcome was the impact of acromion type on therapeutic outcome.

## Search strategy and criteria

Recommendations of the Preferred Reporting Items for Systematic Reviews and Meta-Analyses (PRISMA) statement (Liberati et al. 2009) were completely adopted in our present study.

Three independent authors (LS, LM and PZ) performed a systematic electronic search in PubMed, Embase, Web of Science and the Cochrane Library from inception through February 2016, using the following keywords: “acromioplasty”, “subacromial decompression”, “rotator cuff”, “repair”, “arthroscopic”, “shoulder”, “randomized controlled trials”. In addition, we also searched the ClinicalTrials.gov registry (https://clinicaltrials.gov/) and manually checked the meeting archives of the American Orthopaedic Society for Sports Medicine from 2009 to 2015. Furthermore, identified review articles were systematically assessed, in order to seek additional literatures according to their bibliographies. No language limitations were applied. In total, 99 citations were preliminarily identified after duplicate checking in this process.

**Fig. 1:**
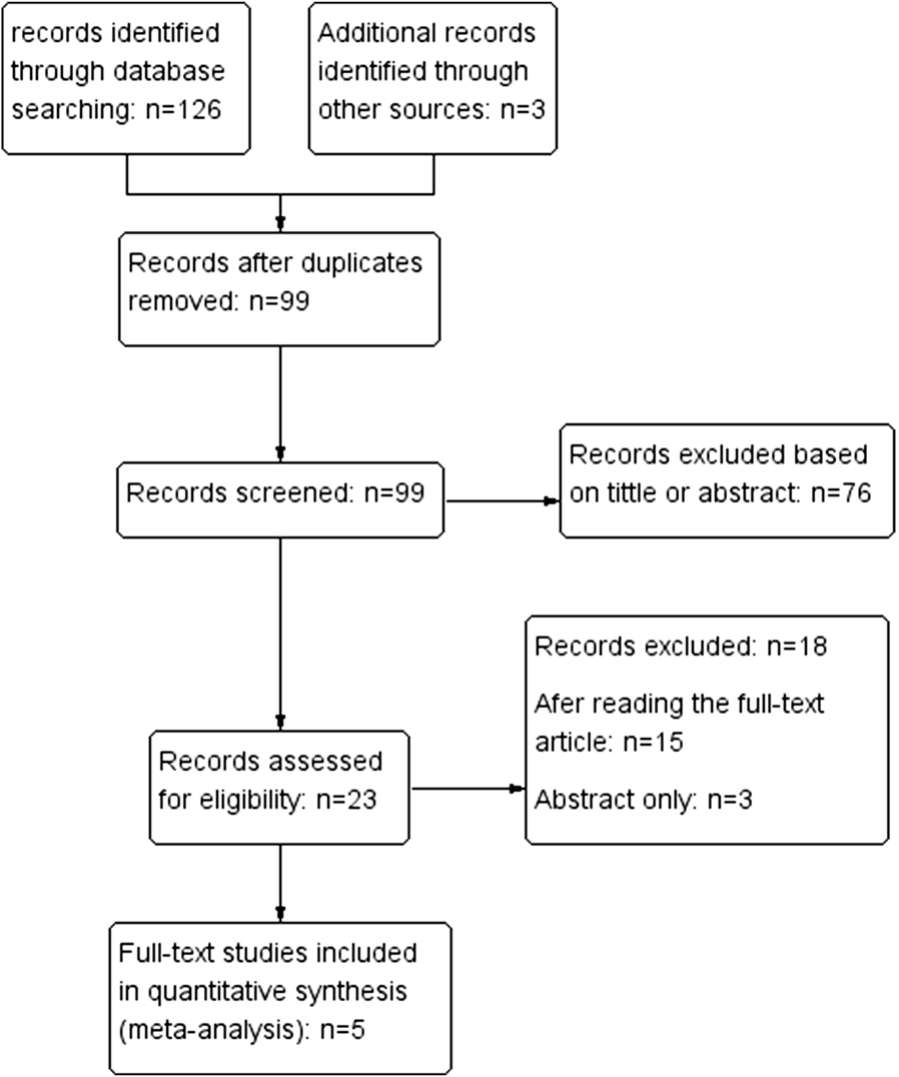
The study flowchart for our literature search is shown

The ultimate purpose of our search was to identify and include all RCTs (level I or II) which were on the role of acromioplasty in arthroscopic repair of full-thickness rotator cuff tears, with a minimum follow-up of one year. To be included, trials had to meet the following criteria: (1) RCTs with comparison in arthroscopic repair of full-thickness rotator cuff tears with and without concomitant acromioplasty; (2) including reports of functional scores and or rate of revision surgery; (3) the least follow-up time was one year; (4) published in full text form. Two reviewers (LS and LM) independently evaluated the retrieved literatures to determine whether they absolutely met the inclusion criteria. A third reviewer (WLW) was included to reach a consensus when necessary. Eventually, five studies (Gartsman and O’connor 2004; Milano et al. 2007; MacDonald et al. 2011; Shin et al. 2012; Abrams et al. 2014) involving 523 patients were available for the present meta-analysis (Fig. 1).

Data extraction was initially performed by LS and confirmed independently by other two reviewers (LM and PZ). The collected characteristics included first author, year of publication, level of evidence, country, inclusion criteria, sample size (the percentage of male patient), patients at follow-up, mean age, mean follow up time, outcome measures and related scores, rate of re-tear (Table 1). Any discrepancy was resolved by discussion until all the authors reached a consensus.

**Table 1:** Characteristics of included studies

References	Level of evidence	Country	Inclusion criteria	Sample size (male %)	Patients at follow-up		Mean age (years)	Mean follow-up (months)	Outcome measures and scores		Re-tear rate	
					A	NA			A	NA	A	NA
Gartsman and O’connor (2004)	I	United States	Isolated, repairable full-thickness supraspinatus tendon tear and type 2 acromion	93 (55)	47	46	59.7	15.6	ASES: 91.5 ± 10.3	ASES: 89.2 ± 15.1	–	–
Milano et al. (2007)	I	Italy	Full-thickness rotator cuff tear and type 2 or 3 acromion	80 (55)	34	37	60.4	24	Constant: 103.6 ± 17.5 DASH: 18.2 ± 17.6 Work-DASH: 23.7 ± 25.3	Constant: 96.1 ± 20.9 DASH: 23.1 ± 19.3 Work-DASH: 26.2 ± 24.8	–	–
MacDonald et al. (2011)	I	Canada	Full-thickness rotator cuff tear ≤4 cm and type 1, 2, or 3 acromion	86 (65)	32	36	56.8	24	ASES: 90.5 ± 13.4 WORC: 87.5 ± 15.3	ASES: 85.6 ± 19.1 WORC: 80.7 ± 21.3	0	4
Shin et al. (2012)	II	South Korea	Full-thickness rotator cuff tear ≤3 cm and type 1, 2, or 3 acromion	150 (56)	60	60	56.8	35	ASES: 90.7 ± 13.1 Constant: 85.0 ± 11.3 UCLA: 33.4 ± 3.3 VAS: 1.1 ± 0.9	ASES: 87.5 ± 12.0 Constant: 83.3 ± 13.0 UCLA: 32.3 ± 3.5 VAS: 1.3 ± 1.4	10	12
Abrams et al. (2014)	II	United States	Full-thickness rotator cuff tear (intra-operative mean tear size) = 2.58 cm and type 1, 2, or 3 acromion	114 (67)	52	43	58.8	24	ASES: 89.0 ± 16.4 Constant: 78.7 ± 11.1 UCLA: 17.4 ± 3.3 VAS: 1.0 ± 1.7 SST: 10.5 ± 2.3	ASES: 91.5 ± 13.3 Constant: 75.0 ± 15.0 UCLA: 17.2 ± 3.4 VAS: 0.7 ± 1.2 SST: 10.5 ± 2.1	1	4

A: acromioplasty group; NA: nonacromioplasty group; –: unclear; ASES: American Shoulder and Elbow Surgeons score; DASH: Disabilities of the Arm, Shoulder, and Hand questionnaire; SST: Simple Shoulder Test; UCLA: University of California-Los Angeles score; VAS: Visual Analog Scale for pain; WORC: Western Ontario Rotator Cuff Index; Work-DASH: Work-Disabilities of the Arm, Shoulder, and Hand questionnaire

**Fig. 2:**
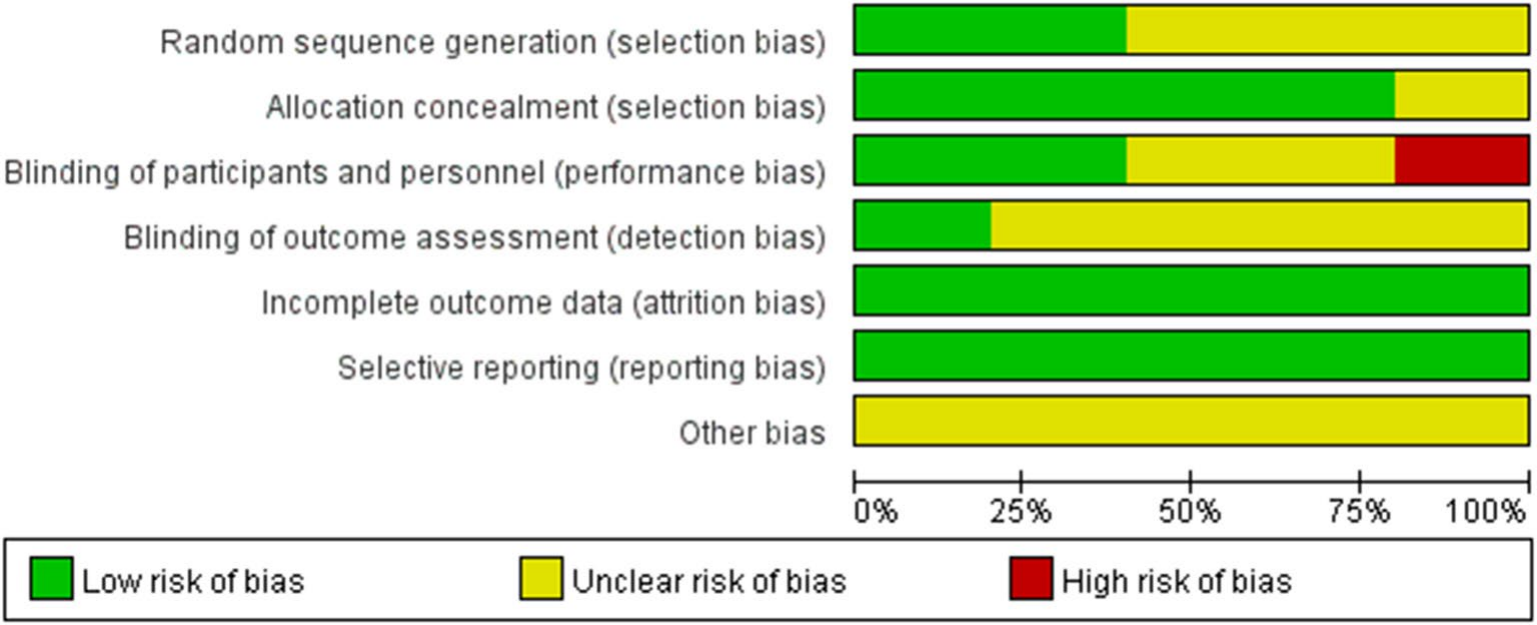
The risk of bias graph shows judgements about each risk of bias item for each included study. + = low risk of bias; – = high risk of bias; ? = unclear or unknown risk of bias

**Fig. 3:**
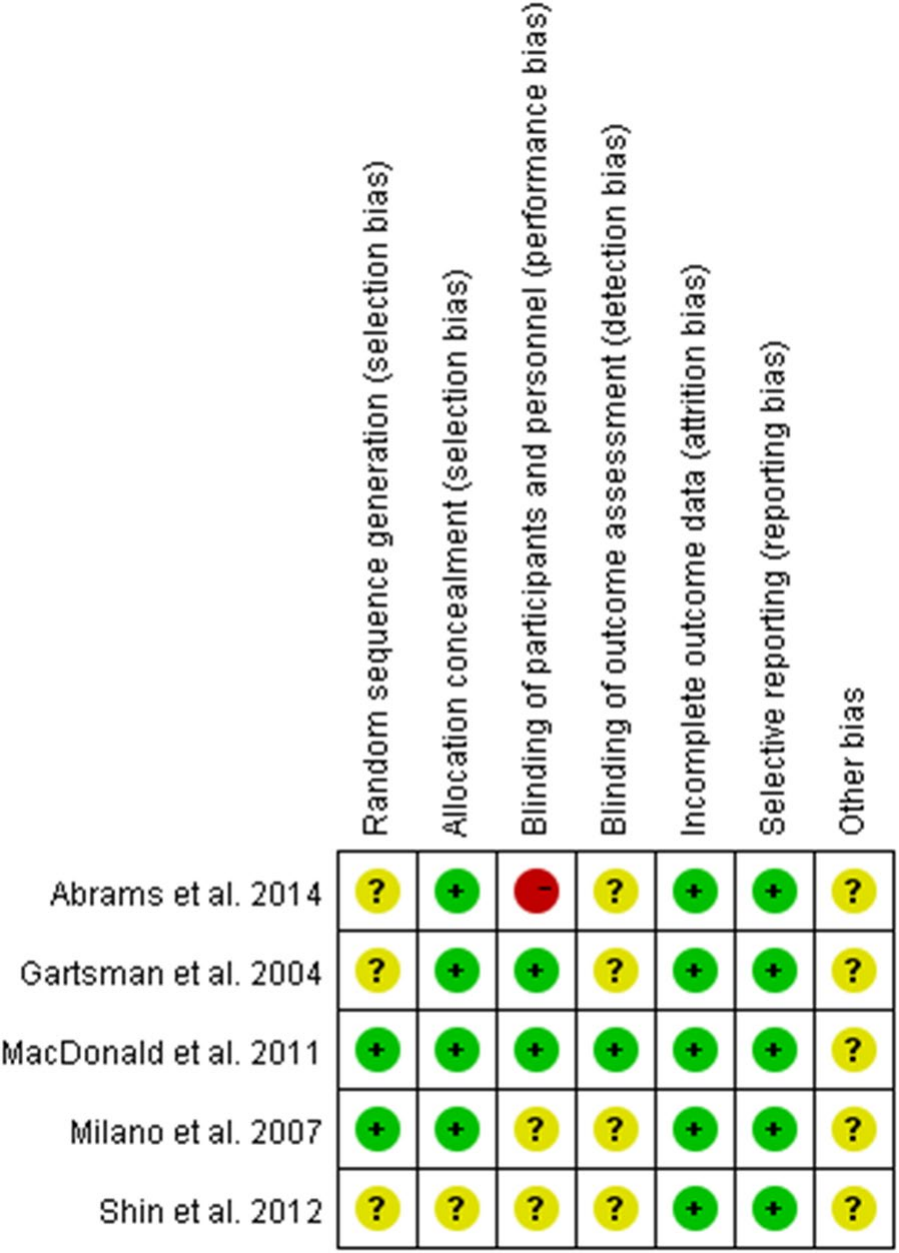
The risk of bias summary shows judgements about each risk of bias item presented as percentages across all included studies

As with our previous published article (Zhang et al. 2015), two reviewers (LS and PZ) independently assessed the risk of bias of eligible studies using the Cochrane risk-of-bias tool (Higgins et al. 2011). Trials included were carefully reviewed and scored as “high”, “low”, or “unclear” risk of bias according to the following criteria: random sequence generation; allocation concealment; blinding of participants and personnel; blinding of outcome assessment; incomplete outcome data; selective reporting; and other bias. Of all the included trials, random sequence generation was explicitly introduced and conducted in two studies (Milano et al. 2007; MacDonald et al. 2011), while the remaining three studies (Gartsman and O’connor 2004; Shin et al. 2012; Abrams et al. 2014) did not adequately introduce the method of randomization, which was only mentioned in the abstracts. Allocation concealment was definitely adopted in four studies (Gartsman and O’connor 2004; Milano et al. 2007; MacDonald et al. 2011; Abrams et al. 2014) via different ways, such as sealed envelopes and codes for assignment. Only one study (Shin et al. 2012) presented unclear in this domain. With respect to blinding of participants and personnel, two studies (Gartsman and O’connor 2004; MacDonald et al. 2011) scored as “low” risk of bias as they had conducted blinding clearly and two studies (Milano et al. 2007; Shin et al. 2012) shared no concrete information. And, more remarkable, the last one (Abrams et al. 2014) presented high risk for disclosing the assignment to the patients. As to blinding of outcome assessment, only one study (MacDonald et al. 2011) showed low risk and in the other remaining studies (Gartsman and O’connor 2004; Milano et al. 2007; Shin et al. 2012; Abrams et al. 2014), it was unclear whether such blinding took place. In terms of incomplete outcome data and selective reporting, all five studies (Gartsman and O’connor 2004; Milano et al. 2007; MacDonald et al. 2011; Shin et al. 2012; Abrams et al. 2014) suggested low risk after repeated scrutiny. No other apparent bias was revealed among the eligible studies (Gartsman and O’connor 2004; Milano et al. 2007; MacDonald et al. 2011; Shin et al. 2012; Abrams et al. 2014) (Figs. 2, 3). In addition, three studies (Gartsman and O’connor 2004; Milano et al. 2007; Shin et al. 2012) did not mention any involved financial support, one study (MacDonald et al. 2011) received funding from a university, and the remaining study (Abrams et al. 2014) stated one or more of the authors had declared the potential conflict of interest or source of funding, indicating that they obtained a certain amount of support.

**Fig. 4:**
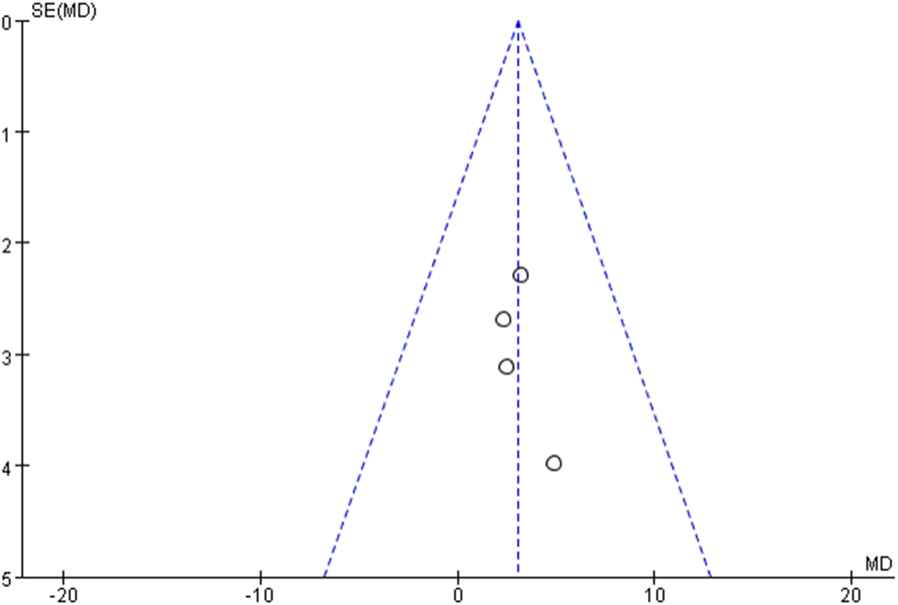
The funnel plot shows the standard error (SE) and mean difference (MD) for the ASES score. *ASES* American Shoulder and Elbow Surgeons

All statistical analyses were performed by two different reviewers (LS and WLW) using Review Manager Software (RevMan5.3; The Nordic Cochrane Centre, The Cochrane Collaboration, Copenhagen, Denmark). Relative risks (RRs) with 95 % CIs were calculated for dichotomous outcomes and weighted mean differences (WMDs) with 95 % CIs for continuous outcomes. The I^2^ statistic was quantified for heterogeneity across studies, and it indicated significant heterogeneity if the I^2^ > 50 % (Higgins et al. 2003). We used a random-effects model for data synthesis with regarding to clinical heterogeneity; otherwise, a fixed-effects model was used. Potential publication bias was evaluated by performing a funnel plot from one of the primary outcomes, ASES score, which was available in four studies (Fig. 4). The studies were distributed along the plot, suggesting that there was no adequate evidence of publication bias. Be that as it may, the assessment was not accurate for a small number of eligible trials. By the same token, a further sensitivity analysis could not be performed to evaluate the latent effects of bias. Thus, we re-ran the searches for potential missing literatures.

To adjust the accompanying increase of type I error (false positive error) of interim analyses in a single randomized clinical trial, monitoring boundaries can be applied to decide whether the trial should be ended timely when a small enough P value appears to indicate the expected effect or certain futility (Goldman and Hannan 2001). Likewise, in a meta-analysis involving repeated significance testing on accumulating data or with a small number of trials, there are some risks yielding the type I error that causes spurious findings (Brok et al. 2009). Therefore, analogous trial sequential monitoring boundaries can also be applied to analyze the pooled results of meta-analysis (Brok et al. 2008). The aforementioned trial sequential monitoring boundaries are concluded from a methodology called trial sequential analysis or named TSA, which can determine the reliability and conclusiveness of the evidence in a meta-analysis. A quantified required information size (RIS) was of paramount importance for the realization of TSA. We estimated the RIS using α = 0.05 (two sided), β = 0.20 (power 80 %) and the empirical data autogenerated from the TSA software (version 0.9 beta, available at http://www.ctu.dk/tsa/) according to the continuous data input. If the cumulative Z-curve crosses the monitoring boundary or the futility boundary, we could draw a credible conclusion that the expected intervention effect might have reached or this intervention has no effect on focused outcome, thereby suggesting further research is not needed even though the RIS line has not been surpassed. When the Z-curve crosses none of the two boundaries and the RIS line, evidence is relatively insufficient to draw a conclusion.

## Results

### Primary outcome: functional scores

Of all the functional scores described in the five included studies (Table 1), only four patient-reported outcomes (ASES score, Constant score, UCLA score and VAS) could respectively be pooled to perform data analysis.

**Fig. 5:**
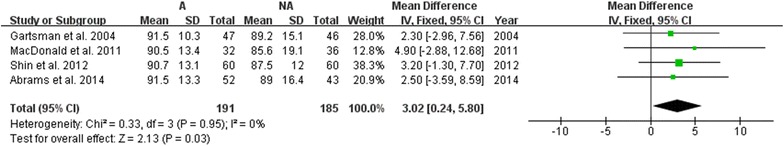
The forest plot of comparison for ASES score between the two different groups. *ASES* American Shoulder and Elbow Surgeons, *A* acromioplasty group, *NA* nonacromioplasty group

**Fig. 6:**
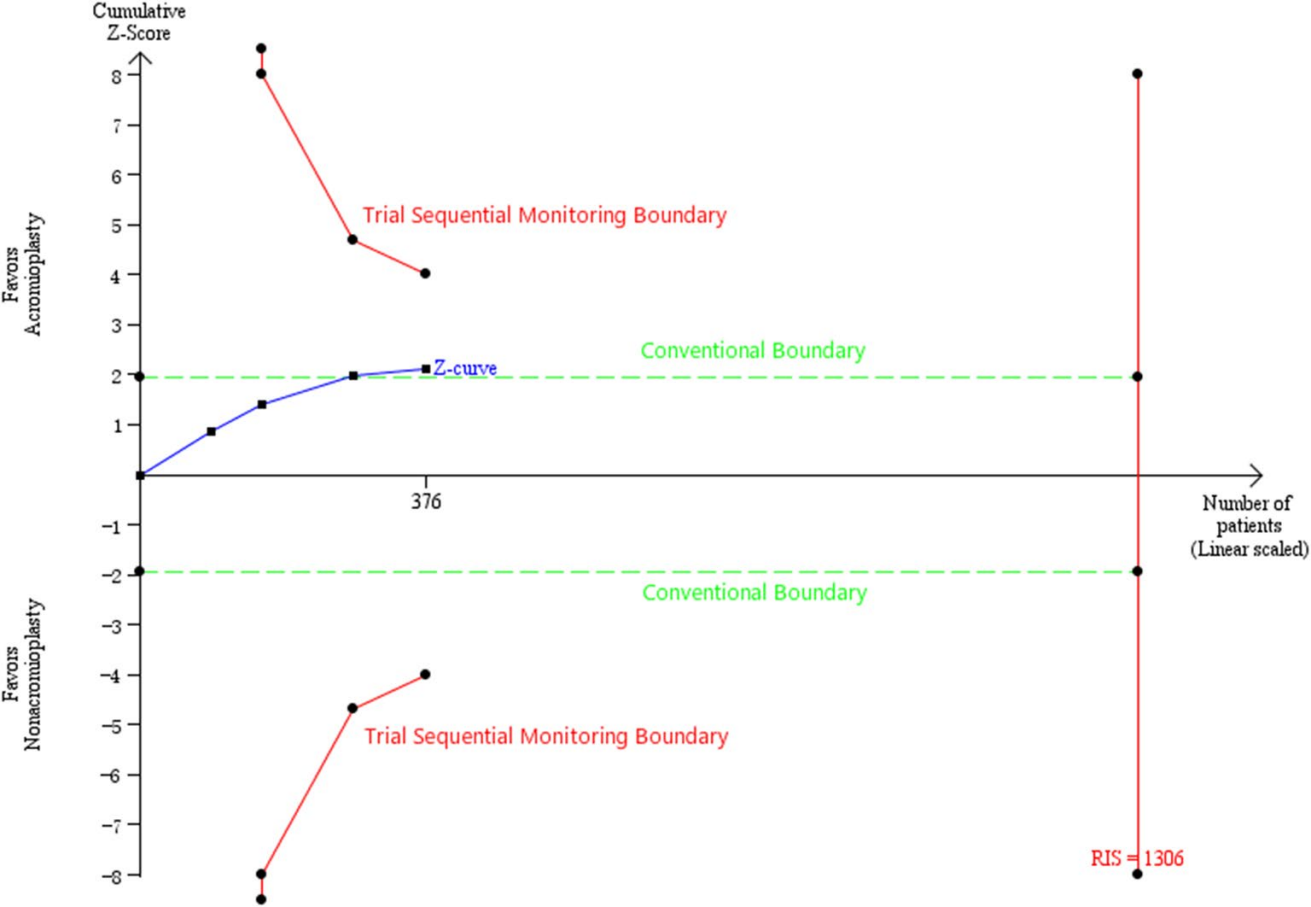
TSA on pooled result of ASES score is shown. The RIS of 1306 patients was estimated using α = 0.05 (two sided), β = 0.20 (power 80 %) and the empirical data autogenerated from software according to the data input. The cumulative Z-curve crossed the conventional boundary, but neither the trial sequential monitoring boundary nor the RIS line was surpassed. *RIS* required information size, *TSA* trial sequential analysis, *ASES* American Shoulder and Elbow Surgeons

Four studies (Gartsman and O’connor 2004; MacDonald et al. 2011; Shin et al. 2012; Abrams et al. 2014) including 376 patients reported the ASES score. A meta-analysis of these trials showed that concomitant acromioplasty was related to a greater increase in ASES score compared with nonacromioplasty among patients undergoing arthroscopic repair of full-thickness tears of the rotator cuff (WMD = 3.02; 95 % CI 0.24–5.80; P = 0.03). No heterogeneity was observed (P = 0.95; I^2^ = 0 %), so we used a fixed-effects model for this analysis (Fig. 5). TSA was conducted at the level of α of 0.05, β of 0.2, and calculated RIS of 1306. Although the Z-curve crossed the conventional statistically significant boundary, it demonstrated that there was no sufficient evidence to draw a definitive conclusion because neither the trial sequential monitoring boundary nor the RIS line was surpassed (Fig. 6).

**Fig. 7:**
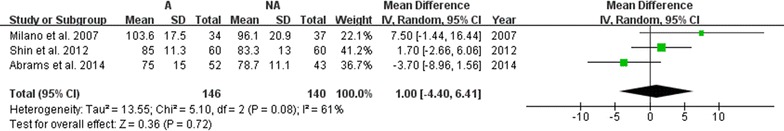
The forest plot of comparison for Constant score between the two different groups. *A* acromioplasty group, *NA* nonacromioplasty group

Three trials (Milano et al. 2007; Shin et al. 2012; Abrams et al. 2014) consisting of 286 patients presented the Constant score. We found no difference between arthroscopic rotator cuff repairs with acromioplasty and those without it according to data synthesis (WMD = 1.00; 95 %CI −4.40 to 6.41; P = 0.72). A random-effects model was used because of moderate heterogeneity (P = 0.08; I^2^ = 61 %) (Fig. 7).

**Fig. 8:**
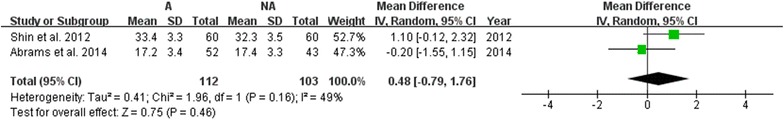
The forest plot of comparison for UCLA score between the two different groups. *UCLA* University of California-Los Angeles, *A* acromioplasty group, *NA* nonacromioplasty group

Two studies (Shin et al. 2012; Abrams et al. 2014) containing 215 patients revealed the UCLA score. No significant difference was seen whether acromioplasty was concomitantly performed or not during arthroscopic repair of full-thickness rotator cuff tears (WMD = 0.48; 95 %CI −0.79 to 1.76; P = 0.46) with moderate heterogeneity (P = 0.16; I^2^ = 49 %), using a random-effects model (Fig. 8).

**Fig. 9:**
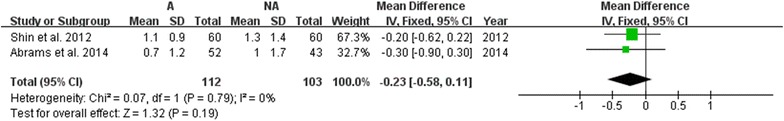
The forest plot of comparison for VAS between the two different groups. *VAS* visual analog scale for pain, *A* acromioplasty group, *NA* nonacromioplasty group

Two studies (Shin et al. 2012; Abrams et al. 2014) of 215 patients disclosed VAS score. We did not see significant difference between the two different surgical procedures (WMD = −0.23; 95 %CI -0.58 to 0.11; P = 0.19). There was no heterogeneity (P = 0.79; I^2^ = 0 %) and we used a fixed-effects model in this comparison (Fig. 9).

### Secondary outcome: rate of re-tear

**Fig. 10:**
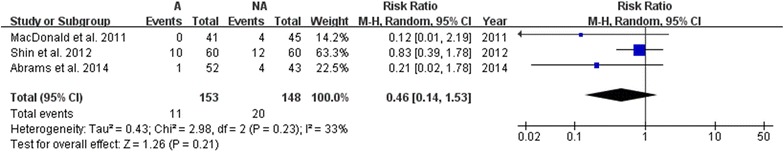
The forest plot of comparison for rate of reoperation between the two different groups. *A* acromioplasty group, *NA* nonacromioplasty group

Including three (MacDonald et al. 2011; Shin et al. 2012; Abrams et al. 2014) studies, which described details of the need for repeated surgery, a meta-analysis was performed. Although one study (MacDonald et al. 2011) reported a higher re-tear rate in patients treated without concomitant acromioplasty, the quantitative synthesis indicated that there was no significant difference between patients treated with acromioplasty and those treated without it during repair of full-thickness tears of the rotator cuff (RR = 0.46; 95 % CI 0.14 to 1.53; P = 0.21). Moderate heterogeneity was detected (P = 0.23; I^2^ = 33 %), therefore, we used a random-effects model in this meta-analysis (Fig. 10).

### Tertiary outcome: impact of acromion type on therapeutic outcome

**Fig. 11:**
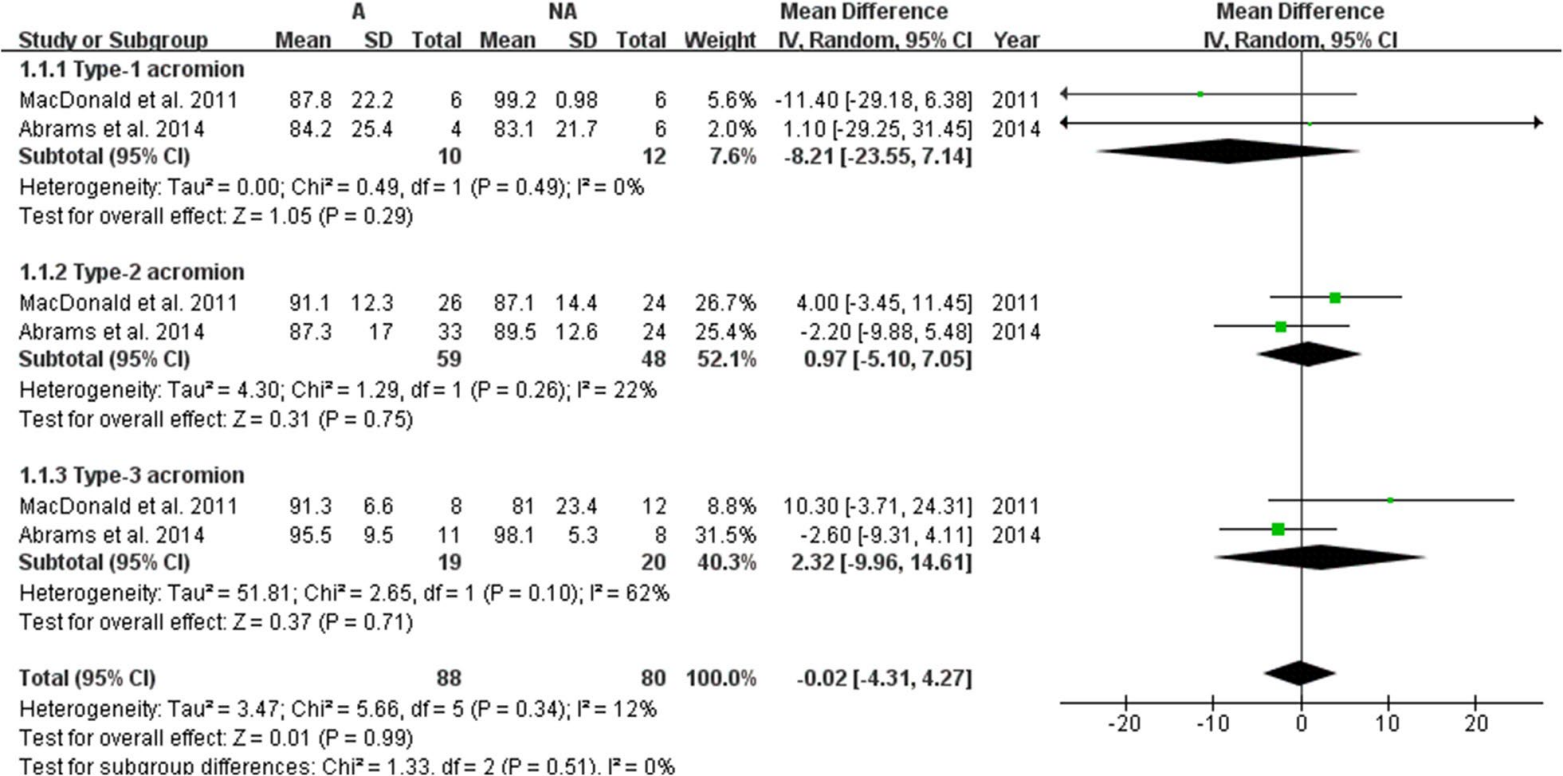
The forest plot of comparison for the impact of acromion type on therapeutic outcome (ASES score) between the two different groups. *ASES* American Shoulder and Elbow Surgeons, *A* acromioplasty group, *NA* nonacromioplasty group

Two RCTs (MacDonald et al. 2011; Abrams et al. 2014) reported the 2-year follow-up ASES score of individuals with various types (1–3) of acromion postoperatively. Subgroup analysis showed no substantial promotion effect of acromioplasty on patients with type-1 acromion (WMD = −8.21; 95 % CI −23.55 to 7.14; P = 0.29), type-2 acromion (WMD = 0.97; 95 % CI −5.10 to 7.05; P = 0.75), and type-3 acromion (WMD = 2.32; 95 %CI −9.96 to 14.61; P = 0.71) as well in regarding to ASES score. No significant heterogeneity was detected in either type-1 acromion subgroup (P = 0.49; I^2^ = 0 %,) or type-2 acromion subgroup (P = 0.26; I^2^ = 22 %,). But, statistically evident heterogeneity was presented in subgroup type-3 acromion (P = 0.10; I^2^ = 62 %). Therefore, a random-effects model was applied (Fig. 11).

## Discussion

The theory of extrinsic subacromial impingement (Neer 1972), which indirectly suggested that acromial morphology was a key initiator in rotator cuff tears, shaped the rationale for the application of concomitant acromioplasty in arthroscopic repair of full-thickness rotator cuff tears. Despite the prevalence of this procedure rising substantially over the past several decades (Vitale et al. 2010; Yu et al. 2010; Paloneva et al. 2015), there is always a controversy surrounding the indeed benefits of acromioplasty in conjunction with rotator cuff repairs. Opponents back up the intrinsic theory that rotator cuff tendons undergo degeneration through aging and overuse (Ozaki et al. 1988; Milgrom et al. 1995; Budoff et al. 1998). Grounding on two RCTs (Gartsman and O’connor 2004; Milano et al. 2007), which were also included in our meta-analysis, the American Academy of Orthopaedic Surgeons gave acromioplasty a “moderate” recommendation (Pedowitz et al. 2011), proposing that “routine acromioplasty is not required at the time of rotator cuff repair”. Besides, acromion which was done acromioplasty could be thicker again after several years postoperatively and the effect of subacromial decompression might be gone with the time. Despite the recent evidence that acromioplasty does not improve rotator cuff healing, surgeons still perform acromioplasty, although less aggressively, because it improves visualization during rotator cuff repair in patients with big spurs and stenotic subacromial space.

In the present meta-analysis, we conducted a thorough quantitative analysis of the available level I and level II evidence to identify the additional value of performing an acromioplasty during arthroscopic rotator cuff repairs. In general, our findings were in accordance with the aforementioned recommendation. There was no significant difference in Constant score, UCLA score, VAS and the re-tear rate in individuals treated with or without concomitant acromioplasty. With regards to functional score, Constant score weigh much on muscle power, and UCLA score is well known as not reliable functional evaluation tool in many reports. Subgroup analysis also found no significant effect of acromial type on ASES score. But notably, a statistically significant difference was found in data synthesis of ASES score (P = 0.03), indicating concomitant acromioplasty was associated with a higher score in this domain. Nevertheless, the TSA result of ASES score suggested insufficient evidence to draw a conclusion.

Objectively speaking, there were some limitations in our meta-analysis. Firstly, methodologic limitations did exist in the included studies. Of all the five RCTs, only one study (MacDonald et al. 2011) used blinding of outcome assessors and patients as well. And even worse, the potential effect of bias could not be evaluated by performing a further sensitivity analysis for small number of studies, although the result of a funnel plot for ASES score showed no apparent publication bias among four related RCTs. Future literatures focused on this topic are needed to settle the uncertainties of our meta-analysis. Secondly, the size of full-thickness rotator cuff tears might be an inevitable effect factor in the course of treatment. One of the included studies (Shin et al. 2012) focused merely on small (<1 cm) and medium-sized (1–3 cm) full-thickness rotator cuff tears, while the other four trials were mixed population of patients in terms of tear size. Thirdly, acromial spurs at the anterior and lateral edges are associated with full-thickness rotator cuff tears (Mihata et al. 2016). Only one study (Shin et al. 2012) stated that the included patients were without acromial spur, but the remaining four study did not refer to it. Therefore, we could not perform the subgroup analyses based on the tear size and acromial spur to ensure the stringency of this meta-analysis. Additionally, various patient groups, clinical settings, suture-and-knotting techniques, operator experiences and postoperative rehabilitation protocols were included in the eligible trials. Thus, data synthesis had a potential risk of introducing significant heterogeneity and study outcome might ultimately be affected. Last but not least, a relatively limited duration of follow-up (15.6–35 months) was observed in the included studies. So, long-term follow-up is needed to consolidate the current findings.

To determine the benefits of concomitant acromioplasty in the treatment of full-thickness rotator cuff tears, postoperative functional outcome scores are important elements. As different scoring systems might result in variations in the objective assessment, we counted all the available scoring systems. The pooled results found no significant difference in Constant score, UCLA score, VAS in the comparisons. Nevertheless, the correlation between the increased ASES score and concomitant acromioplasty was also observed, and a further TSA result of ASES score suggested insufficient evidence. So, the correlation between the increased ASES score and concomitant acromioplasty could not be absolutely confirmed and more evidence was needed with regard to the result of TSA. In terms of the above inconsistence of various score, we could not draw a definite conclusion on this topic.

Simultaneously, orthopaedists draw much attention to the postoperative rate of re-tear. Using a dynamic shoulder model, Wuelker et al. (1995) proved that mean coracoacromial pressures decreased 5 % after performing anterior acromioplasty. Moreover, Bigliani et al. (1992) indicated that a major cause of rotator cuff repair failure was insufficient subacromial decompression. One (Shin et al. 2012) included study reported a significantly higher rate in the number of patients requiring repeated surgery for the nonacromioplasty group. And there were also more patients requiring additional surgery in the nonacromioplasty group in the other two studies (MacDonald et al. 2011; Abrams et al. 2014). 11 of 153 patients in acromioplasty group and 20 of 148 individuals in nonacromioplasty group who needed repeated surgery were included in our meta-analysis and the result indicated that there was no statistically significant difference between the two groups.

Another major concern lies in the acromion morphology. In a cadaveric study (Bigliani et al. 1991), 70 % of specimens with a type-3 hooked acromion and an anterior spur were found to have full-thickness rotator cuff tears. Balke et al. (2013) conducted a radiographic study and indicated that extremely hooked anterior acromion with a slope of more than 43° and an lateral acromial angle of less than 70° occurred exclusively in individuals with rotator cuff tears. In our meta-analysis, three (MacDonald et al. 2011; Shin et al. 2012; Abrams et al. 2014) of the included studies enrolled patients with all the three types of acromion. However, in the remaining two studies, one (Gartsman and O’connor 2004) included only type-2 acromion, while the other (Milano et al. 2007) included both type-2 and type-3 acromion. Three (Milano et al. 2007; MacDonald et al. 2011; Abrams et al. 2014) of the five eligible studies showed acromion type had no significant effect on postoperative functional scores. To take it a step further, with an underpowered subgroup analysis, Abrams et al. (2014) reported that a type-3 acromion had a negative effect on the Constant score, SST score, and VAS compared with a type-1 acromion. However, in our study, subgroup analysis indicated that various types (1–3) of acromion did not have apparent association with higher ASES score, Although the other functional scores were not available to be pooled in this domain. Similarly, rate of re-tear was highly suspected to be connected with acromion morphology. Despite finding that three of the four patients who suffered repeated surgery had a type-3 acromion in one study (MacDonald et al. 2011), we were unable to perform a formal meta-analysis of this topic for lacking of productive data. Thereby whether acromion morphology has effects on the rate of re-tear can not be elucidated yet.

Compared to the pervious meta-analysis (Chahal et al. 2012) on this topic, several differences should be noted in ours. First, the present meta-analysis showed inconsistent result of pooled ASES score, even if, of note, we could not substantiate the finding with further applied TSA. Next, the previous meta-analysis totaling 373 patients included three published RCTs (Gartsman and O’connor 2004; Milano et al. 2007; MacDonald et al. 2011) and one preliminary result reported in abstract form only, whereas our present meta-analysis included five published RCTs (Gartsman and O’connor 2004; Milano et al. 2007; MacDonald et al. 2011; Shin et al. 2012; Abrams et al. 2014) and 523 individuals, which to some extent reinforced earlier results of previous meta-analysis in pooled Constant score and the rate of re-tear. Furthermore, we took additional evidence, UCLA score and VAS and even subgroup analysis, into our meta-analysis. Last but not least, as mentioned above, we assessed the evidence for pooled ASES score using TSA to facilitate the implementation of corresponding clinical decisions of healthcare professionals.

## Conclusion

Our present meta-analysis indicated that arthroscopic repair of full-thickness rotator cuff tears with acromioplasty, compared with that without it, might have a higher ASES score, despite further TSA suggested insufficient evidence to draw a conclusion. However, no difference was found in Constant score, UCLA score, VAS and rate of re-tear as well during the comparisons. Additionally, subgroup analysis did not found substantial impact of acromion type on the eventual outcome, available with ASES score only. Therefore, further well-designed clinical studies with large number of participants, long-term follow-ups, data of patient-reported outcomes and stratification for acromion type are of the essence for confirming whether functional or structural differences exist in patients undergoing arthroscopic repair of full-thickness rotator cuff tears with or without acromioplasty.

## Abbreviations

RCTs: randomized controlled trials
TSA: trial sequential analysis
CAL: coracoacromial Ligament
SAD: subacromial decompression
A: acromioplasty group
NA: nonacromioplasty group
ASES: American Shoulder and Elbow Surgeons score
DASH: Disabilities of the Arm, Shoulder, and Hand questionnaire
SST: simple shoulder test
UCLA: University of California-Los Angeles score
VAS: visual analog scale for pain
WORC: Western Ontario Rotator Cuff Index
Work-DASH: Work-Disabilities of the Arm, Shoulder, and Hand questionnaire
SE: standard error
MD: mean difference
WMD: weighted mean difference
